# The effects of dietary linoleic acid and hydrophilic antioxidants on basal, peak, and sustained metabolism in flight‐trained European starlings

**DOI:** 10.1002/ece3.6010

**Published:** 2020-01-18

**Authors:** Wales A. Carter, Kristen J. DeMoranville, Barbara J. Pierce, Scott R. McWilliams

**Affiliations:** ^1^ Department of Natural Resources Science University of Rhode Island Kingston RI USA; ^2^ Department of Biology Sacred Heart University Fairfield CT USA

**Keywords:** antioxidants, BMR, fatty acids, flight training, PMR, songbirds, sustained metabolism

## Abstract

Dietary micronutrients have the ability to strongly influence animal physiology and ecology. For songbirds, dietary polyunsaturated fatty acids (PUFAs) and antioxidants are hypothesized to be particularly important micronutrients because of their influence on an individual's capacity for aerobic metabolism and recovery from extended bouts of exercise. However, the influence of specific fatty acids and hydrophilic antioxidants on whole‐animal performance remains largely untested. We used diet manipulations to directly test the effects of dietary PUFA, specifically linoleic acid (18:2n6), and anthocyanins, a hydrophilic antioxidant, on basal metabolic rate (BMR), peak metabolic rate (PMR), and rates of fat catabolism, lean catabolism, and energy expenditure during sustained flight in a wind tunnel in European starlings (*Sturnus vulgaris*). BMR, PMR, energy expenditure, and fat metabolism decreased and lean catabolism increased over the course of the experiment in birds fed a high (32%) 18:2n6 diet, while birds fed a low (13%) 18:2n6 diet exhibited the reverse pattern. Additionally, energy expenditure, fat catabolism, and flight duration were all subject to diet‐specific effects of whole‐body fat content. Dietary antioxidants and diet‐related differences in tissue fatty acid composition were not directly related to any measure of whole‐animal performance. Together, these results suggest that the effect of dietary 18:2n6 on performance was most likely the result of the signaling properties of 18:2n6. This implies that dietary PUFA influence the energetic capabilities of songbirds and could strongly influence songbird ecology, given their availability in terrestrial systems.

## Graphical abstract

Metabolic capacity contributes to the ability of songbirds to engage in endurance flight and may be influenced by dietary micronutrients such as fatty acids and antioxidants. We tested the effects of linoleic acid and anthocyanins on songbird metabolism with a diet manipulation experiment. The variable influence of linoleic acid on basal, peak, and sustained metabolism over the experiment suggests that these effects are mediated via linoleic acid's signaling properties and demonstrates the potential impact of diet selection on songbird performance.

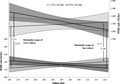

## 1 INTRODUCTION

The success of individual animals depends on their ability to acquire diets with sufficient resources for maintenance, activity, and reproduction, which in turn shapes ecological niches and interactions among species. Besides energy and macronutrient content, the adequacy of animal diets often depends on their micronutrient composition, as demonstrated, for example, by the influence of sodium and calcium on the habitat selection, movements, and population dynamics of mammals and birds (Aumann & Emlen, 1965; Belovsky & Jordan, 1981; Wilkin, Gosler, Garant, Reynolds, & Sheldon, 2009). Dietary fatty acids include essential micronutrients and polyunsaturated fatty acids (PUFA), with demonstrated effects on ecologically relevant performance of a wide range of taxa. For example, dietary PUFA has been found in some studies to affect active and resting metabolic rates in songbirds and humans (Pierce, McWilliams, O’Connor, Place, & Guglielmo, 2005; Twining et al., 2016; van Marken Lichtenbelt, Mensink, & Westerterp, 1997), mitochondrial metabolism in ground squirrels (Gerson, Brown, Thomas, Bernards, & Staples, 2008), aerobic endurance and efficiency in songbirds (McWilliams & Pierce, 2006), and maximal speed in 36 species of mammals (Ruf, Valencak, Tataruch, & Arnold, 2006) and salmon (McKenzie & Higgs, 1998). Results such as these suggest that (a) the fatty acids that are important micronutrients for many wild vertebrates are primarily long‐chain polyunsaturated, likely due to their unique chemical properties and limited synthesis by vertebrates (Klasing, 1998; Twining, Lawrence, Winkler, Flecker, & Brenna, 2018), (b) dietary fatty acids seem to primarily influence the regulation and scope of energy metabolism (Pierce & McWilliams, 2014; Price, 2010), and (c) the effects of dietary fatty acids have particular relevance for birds during migration, possibly due to the high metabolic demands of flight (Guglielmo, 2010; Martinez del Rio & McWilliams, 2016). However, the influence of dietary fatty acids on the performance of migratory birds remains decidedly equivocal, with inconsistent results across different measures of performance (Pierce et al., 2005), across different ages (Amitai, Bauchinger, McCue, & Pinshow, 2009; Price et al., 2018), and across species (Dick & Guglielmo, 2019a; McWilliams & Pierce, 2006; Price & Guglielmo, 2009), including several studies that failed to find any effects. The inconsistency of past results suggests a need for more refined tests of the influence of individual fatty acids on multiple measures of performance.

Dietary antioxidants are also important micronutrients for birds, in general (Klasing, 1998), and for birds in migration, specifically (Alan, McWilliams, & Mcgraw, 2013; Bolser et al., 2013). Functionally, dietary antioxidants act to reduce oxidative damage (Beaulieu & Schaefer, 2013; Catoni, Peters, & Schaefer, 2008; Skrip & McWilliams, 2016), as well as influence immune function (Catoni, Schaefer, & Peters, 2008; Marri & Richner, 2015), investment in reproduction (Royle, Surai, & Hartley, 2003; Skrip, Seeram, Yuan, Ma, & McWilliams, 2016), reproductive hormones and behavior (Carbeck et al., 2018), and ability to meet the oxidative costs of flight (Larcombe et al., 2008; Skrip et al., 2016). However, as with fatty acids, results have differed among studies that used different measures of performance and that supplemented diets with different antioxidants, with hydrophilic antioxidants being largely untested (Cooper‐Mullin & McWilliams, 2016).

Dietary fatty acids and antioxidants may also interact during their metabolism and synergistically affect animal performance. High‐PUFA diets and subsequently enhanced oxidative capacity may be beneficial when energy demands are elevated by long bouts of flight (Dingle, 2014; Wikelski et al., 2003), as when minimizing time on migration (Alerstam & Hedenstrom, 1998). However, elevated metabolism also generally involves the increased production of pro‐oxidants (Jenni‐Eiermann, Jenni, Smith, & Costantini, 2014; Larcombe et al., 2008; Mataix, Quiles, Huertas, Battino, & Mañas, 1998) and the storage of PUFAs increases the risk of lipid peroxidation (Hulbert, 2010; Skrip et al., 2015; Skrip & McWilliams, 2016). Thus, songbirds must balance the need to meet high metabolic demands with the risk of oxidative damage, and this tradeoff may be mitigated with the intake of dietary antioxidants. Additionally, migratory songbirds undergo a range of physiological changes in preparation for migration including the upregulation of lipid transporters, oxidative enzymes, and hormones (Corder, DeMoranville, Russell, Huss, & Schaeffer, 2016; McFarlan, Bonen, & Guglielmo, 2009; Price, Bauchinger, et al., 2011), which could interact with dietary fatty acids or antioxidants to produce changes in performance over time.

We experimentally tested how dietary linoleic acid (18:2n6), a leading candidate to mediate the effects of dietary fatty acid composition on metabolism in songbirds (Pierce & McWilliams, 2014; Pierce et al., 2005; Price & Guglielmo, 2009; Price et al., 2018), and the dietary anthocyanins, hydrophilic antioxidants common in the diets of wild songbirds (Bolser et al., 2013; Cooper‐Mullin & McWilliams, 2016), interact to affect exercise performance of European starlings (*Sturnus vulgaris*). We measured performance in terms of metabolic rates and tissue catabolism during sustained flight in a wind tunnel to allow us to assess the effect of diet on basal, peak, and sustained metabolism as well as the relationship between these measures. This study provides one of the first tests of the hypothesis that metabolic rates of songbirds are influenced by specific dietary fatty acids and is the first to test how performance during sustained flight is influenced by the interacting effects of dietary fatty acids and water‐soluble antioxidants. Both of these are key steps toward understanding the potential influence of fatty acid and antioxidant nutrition on the ecology of wild songbirds.

## 2 METHODS

### 2.1 Animal care and experimental diets

This experiment took place from August to December, 2015, at the Advanced Facility for Avian Research (AFAR) at the University of Western Ontario (UWO) in London, ON, CA, and was covered by animal care protocols for UWO (2010–2016) and the University of Rhode Island (AN11‐12‐009). Between 19 and 23 August 2015, we captured 108 hatch‐year European starlings (*S. vulgaris*) at a dairy farm approximately 20 km north of UWO in Middlesex Center, ON (43.17°N, 81.36°W) and immediately transported birds to AFAR where they were housed in four large indoor aviaries (two 2.4 × 3.7 × 3.1 m and two 2.4 × 2.3 × 3.5 m). Migratory behavior has been inferred from band returns in this population (Cabe, 1993), but may not extend to the majority of individuals. On 24 August, we measured the body mass, wing chord, tarsus, culmen, head length, and molt score (0–75; Ginn & Melville, 1983) of each bird and randomly resorted the birds into the four aviaries such that each aviary housed a group of birds with roughly equal body mass and molt score distributions. Aviaries were kept at 21°C and on a natural light cycle for London, ON (14.5 hr light: 9.5 hr dark at capture). Each week, we weighed, measured molt, and visually inspected birds for abnormalities to monitor their health.

Starting at capture, birds in each aviary had ad libitum access to water and one of two isocaloric, semisynthetic diets that differed only in fatty acid (FA) composition (Table 1). Dietary fatty acids were manipulated by including different proportions of canola, sunflower, and palm oil so that the diets were either high (32%) or low (13%) in 18:2n6, which was primarily traded off with palmitic acid (16:0). There were several other much less common fatty acids that differed between diets, but because these differences were much smaller in magnitude we have focused our interpretation on the manipulation of 18:2n6 and 16:0. We lightly supplemented the diet with dried mealworms for three weeks postcapture to facilitate the birds’ transition from natural to experimental diets in captivity. Starting on 1 September, we supplemented the diets of birds in one 32% and one 13% 18:2n6 aviary with dried elderberry powder (Artemis International, Fort Wayne, IN) while making the diets as a source of hydrophilic antioxidants (AOX) at a concentration recommended for poultry being fed a high‐fat diet (30 IU/kg; Scott, Nesheim, & Young, 1982). The vitamin mix added to all diets provided a low baseline level of lipophilic dietary antioxidants (7.6 mg/g dry diet α‐tocopherol). Thus, after 1 September the experimental design was a 2 × 2 factorial with four groups: 32% 18:2n6 high AOX (*N* = 28), 32% 18:2n6 low AOX (*N* = 27), 13% 18:2n6 high AOX (*N* = 27), and 13% 18:2n6 low AOX (*N* = 26).

**Table 1:** Ingredients and fatty acid composition of semisynthetic diets used in this study

Ingredients	% of dry mass	Oil mixture (%)	
		High 18:2n6	Low 18:2n6
Agar^a^	3.19		
Casein^a^	19.12		
Cellulose^a^	4.97		
d‐glucose^b^	39.18		
Amino acid mix^c^	2.68		
Salt mix^d^	4.78		
Vitamin mix^d^	0.38		
Elderberry powder^e^	0.42		
Mealworms^f^	6.16		
Plant oils^g^	19.12		
Fatty acid^h^:			
12:0		0.24	0.21
14:0		0.38	0.91
16:0		8.65	29.64
16:1n7		0.3	0.41
18:0		2.83	3.9
18:1n9		45.2	42.76
18:2n6		31.87	13.86
18:3n3		4.05	2.68
20:1n9		0.55	0.4
20:4n6		0	0
22:6n3		0.15	0.11
24:1		0.1	0.08

a U.S. Biomedical Corp., Cleveland, OH.

b Fisher Scientific, Waltham, MA.

c Assembled after Murphy and King (1982) from individual amino acids supplied by Fisher Scientific.

d MP Biomedicals, Santa Ana, CA.

e High antioxidant diets only: Artemis International, Fort Wayne, IN.

f Freeze dried: Exotic Nutrition, Newport News, VA.

g low 18:2n6 diet: canola oil and palm oil, high 18:n6 diet: canola oil and sunflower oil, supplied by Jedwards International, Braintree, MA.

h Diet fatty acid concentrations (percent by mass) was measured by gas chromatography in lipids extracted from the diets. Only the twelve most concentrated fatty acids are listed.

### 2.2 Cohort assignment and metabolic measurements

On 21 September, we fixed the light schedule at 11:13 L:D and assigned birds in each diet group to one of five cohorts (*N* = 5–6 per cohort) in descending order of molt score (i.e., from most to least advanced in molt) that corresponded to the order in which they would be flight trained in the wind tunnel. The resulting twenty cohorts were then ordered by the average molt score of birds within each cohort. Starting on 23 September, and continuing every three days thereafter (Supporting Information Appendix A: Figure A1), we removed the individuals of the appropriate cohort from their aviary, and then weighed, blood sampled, and measured the body composition (fat and lean mass) of each individual with a quantitative magnetic resonance (QMR; Echo Medical Systems, Houston, TX) instrument calibrated daily with a canola oil standard (Guglielmo, McGuire, Gerson, & Seewagen, 2011). Three of the five individuals in each cohort were then randomly assigned to the flight‐training group, and two were assigned to the untrained control group. All birds in the cohort were then moved to individual 0.6 × 0.5 × 0.5 m cages with ad libitum access to food and water for two days (days −9 and −8 relative to the start of flight training for a given cohort, see below), and on day −7, we measured birds’ food intake.

Starting at 8:00 p.m. on the night of day −6, we used flow‐through respirometry to measure the pretraining basal metabolic rate (BMR) of the three birds assigned to the flight‐training and one control bird. Dried air flowed through four individual chambers housed in an incubator set at 27°C (Wiersma, Salomons, & Verhulst, 2005), and the gas composition of effluent air was measured in a rotation of the background source and individual chambers. Flow rate and partial pressures of oxygen, carbon dioxide, and water vapor were measured with a Sable Systems (Las Vegas, NV) Flowbar‐8 Flow Controller, FC‐1b O_2_ Analyzer, CA‐2A CO_2_ Analyzer, and RH‐300 Water Vapor Analyzer, respectively. Since our questions involved relative comparisons of diet groups, we present BMR as the lowest instantaneous oxygen consumption averaged over a five‐minute period, corrected for time lag, water vapor pressure, and CO_2_ concentration using equations 8.6 and 11.7 in (Lighton, 2008). This period occurred more than six hours after the start of the trial for all birds, supporting the assumption that the birds were postabsorptive.

The following morning (day −5), we measured the peak metabolic rate (PMR) of the same four birds used to measure BMR in a flight‐hover wheel similar to those used by Pierce et al. (2005) and Price and Guglielmo (2009) using the same gas analyzers as above and an MFC‐2 flow controller. The internal width and diameter of the wheel were both 30 cm, and the flow rate of air was 5 L/min. For each trial, we measured the baseline gas concentrations for two minutes before the bird was weighed, introduced to the chamber, and given five minutes to become acclimated. After the acclimation period, the wheel was spun slowly (~30 RPM) for two minutes and then ramped up to a speed where the bird's wings were in constant motion (~60–90 RPM). This pace was held until the bird's oxygen consumption plateaued or declined for at least two minutes, after which the bird was removed and gas concentrations were allowed to return to baseline. We calculated PMR as the highest instantaneous oxygen consumption averaged over a one‐minute period, which occurred 15–30 min after the start of the PMR measurements for all birds. Following PMR measurements, flight‐training birds were moved to a 0.8 × 1.5 × 2 m flight aviary and control birds were returned to their initial aviaries. Control birds did not differ from exercise training birds at the time of metabolic rate measurements and by definition could not be used in subsequent tests of exercise performance that required flight training. The control group was included as part of a broader study on songbird exercise physiology and is only analyzed and discussed in this manuscript in the one case where a meaningful contrast between trained and control birds was possible: tissue fatty acid composition (see below).

### 2.3 Flight training and exercise performance

In order to assess the effect of diet on sustained exercise performance, we put the three flight‐training birds in each cohort through four days of pretraining and a fifteen‐day wind tunnel flight‐training regimen, which has demonstrated success at eliciting long‐duration flights (Engel, Biebach, & Visser, 2006). Pretraining (day −4 to day −1) included training birds to fly between their flight cage and the wind tunnel and twenty minutes of acclimation time per day inside the wind tunnel with a perch available and at progressively increasing wind speeds (0–12 m/s). Flight training consisted of one hour fast and then continuous flight at 12 m/s wind speed, 15°C, and 70% humidity for increasing durations over the course of the training regimen: day 1, 20 min; day 2, 20 min; day 3, 20 min; day 4, 20 min; day 5, 30 min; day 6, 30 min; day 7, 60 min; day 8, 90 min; day 9, 30 min; day 10, 120 min; day 11, 180 min; day 13, 60 min; and day 14, 30 min. Day 12 was a rest day, during which we returned birds to individual cages and measured food intake. At 8:00 a.m. on day 14, we collected a 400 μl blood sample from the brachial vein of each bird for measurements of preflight levels of plasma metabolites. Flight training concluded on day 15 with a flight that started at 8:00 a.m. and lasted as long as birds would voluntarily fly (maximum of 360 min). Body composition was measured with the QMR within 20 min of starting their longest flight. Immediately after the flight, we took a second blood sample for measurements of postflight plasma metabolite levels and again measured the body composition of each bird with the QMR.

We used several metrics to assess the effect of diet on sustained exercise performance during long flights on day 15, including flight duration, the rates of fat and lean tissue catabolism during flight, and the rate of energy expenditure during the flight. Rates of fat and lean tissue catabolism were estimated by dividing the difference between pre‐ and postflight fat and lean masses, obtained from the QMR, by flight duration. The rate of energy expenditure was estimated by multiplying the mass of fat and lean tissue lost during flight by their respective energy densities (39.7 and 17.8 kJ/g; Schmidt‐Nielsen, 1997), adding them, and dividing by fight duration. All raw fat and lean mass measurements were corrected to dry masses following Guglielmo et al. (2011) before calculations.

### 2.4 Tissue fatty acid composition

Untrained control birds and flight‐training birds from a given cohort were euthanized, and samples of all major organs and muscle groups were taken on days 16 and 17, respectively, as part of a larger study on the effects of diet and training on exercise physiology. The sex of each bird was confirmed during dissection. To verify the effect of diet on tissue fatty acid composition, we analyzed the composition of pectoral muscle lipid membranes and intracellular fat droplets extracted using a modified Folch method (Folch, Lees, & Stanley, 1957; Guglielmo, Hara, & Williams, 2002). Briefly, we homogenized approximately 200 mg of wet tissue in 6 ml of 2:1 chloroform:methanol, centrifuged at 3,000 rpm for 15 min, separated aqueous solutes by rinsing with 0.25% KCl, and transferred the organic phase to a glass vial, where it was dried under N_2_ and resuspended in chloroform. We separated whole lipid samples into lipid droplet, nonesterified fatty acid (NEFA), and membrane fractions in solid phase extraction columns (Supelco, LC‐NH_2_, 1ml aminopropyl bonding) with elusions of 2:1 chloroform:isopropanol, 49:1 isopropyl ether:acetic acid, and methanol. We collected lipid droplet and membrane fractions and then esterified them into fatty acid methyl esters (FAMEs) by heating at 90°C for 2 hr in 1M acetyl chloride in methanol. Duplicate 1 µl aliquots of sample FAMEs (1 mg/ml in dichloromethane) were injected into a Shimadzu Scientific Instruments QP2010S GC‐MS linked to a 2010 FID (Shimadzu Scientific Instruments, Kyoto, Japan) at Sacred Heart University (Fairfield, CT). Peaks were identified by retention times established by analysis of GLC standard FAME mixes (Nu‐Chek Prep, Elysian, MN USA) run every 15 samples and visual inspection of all chromatograms. Concentrations of individual FAs were calculated as a percent by mass (FA peak area/total chromatogram area).

### 2.5 Plasma metabolites

To complement fuel use (Jenni‐Eiermann et al., 2002; Skrip et al., 2015; Smith & McWilliams, 2009), we measured the concentrations of triglycerides, uric acid, and β‐hydroxybutyrate in the pre‐ and postflight plasma samples taken on day 15. Blood samples were collected in capillary tubes, centrifuged at 5,000 rpm for 10 min, and plasma was separated and stored at −80°C until analysis. We assayed metabolite concentrations on 96‐well plates with a Biotek Synergy HTX plate reader (Biotek Instruments, Winooski, VT) using commercial kits adapted for use with small volumes. Triglycerides and uric acid were diluted 1:1 with 0.9% saline and measured using an absorbance endpoint assays (Sigma‐Aldrich, St. Louis, MO and TECO Diagnostics, Anaheim, CA, respectively). β‐hydroxybutyrate was diluted 1:4 with tris buffer (pH 8.5) and measured using an absorbance endpoint assay (Cayman Chemical, Ann Arbor, MI). All assays were run in duplicate, and concentrations are presented mmol/L.

### 2.6 Statistical analyses

All statistical analyses were completed in R (v3.3.2; R Core Team, Vienna, Austria). We analyzed metabolic data (*N* = 76) using linear models that tested the effects of dietary FA, dietary AOX, date, and sex, and we included body mass as a covariate for both BMR and PMR and time of day as a covariate for PMR. Similarly, we used linear models with the above effects and preflight fat mass to describe the exercise performance of birds who completed flights longer than two hours (*N* = 43), the time necessary for the total amount of tissue catabolized at the average rate of tissue loss–1 *SD* to exceed the error in QMR measurements. There were no discernable patterns across the seven birds that failed to reach this cutoff, which were evenly spread across diet and from the first to last cohort. We also tested for effects of diet and date on body composition (preflight fat and lean masses). We used a Hotelling's *t* test to compare overall fatty acid composition between 32% and 13% groups and tested for correlations between the composition of 18:2n6 and metabolic rates and exercise performance. Finally, we compared log‐transformed pre‐ and postflight plasma metabolite levels with linear mixed models including time (pre‐ or postflight), date, and diet as fixed effects and individual as a random effect. Degrees of freedom for linear mixed models were obtained using a Satterthwaite approximation.

## 3 RESULTS

### 3.1 Metabolic rates

**Figure 1:**
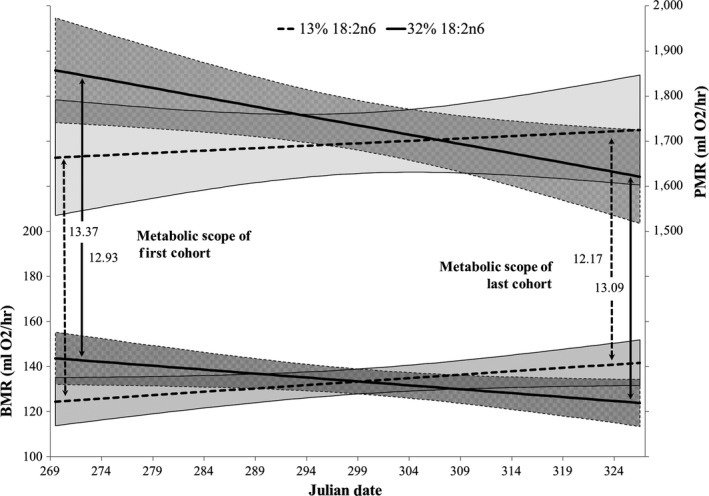
Basal (BMR) and peak (PMR) metabolic rates of starlings fed either high (32% of total FA, *N* = 40) or low (13%, *N* = 36) amounts of 18:2n6. Metabolic scope refers to the quotient of PMR/BMR, and shaded areas indicate 95% confidence intervals of regressions. Julian date 269 to 324 is 27 September to 20 November 2015 and so is 26 to 81 days after being introduced to the four experimental diets on 1 September

We found a consistent relationship between metabolic rates, date, and dietary FA composition (Figure 1, Supporting Information Appendix B: Table B‐1): The metabolic rates of birds fed the 32% diet started high and decreased over successive cohorts, while those of birds fed the 13% diet started low and increased over successive cohorts (Diet × Date interaction: BMR: *T*_69_ = 3.228, *p* = 0.002; PMR: *T*_68_ = 2.118, *p* = 0.038). PMR significantly increased with body mass (*T*_68_ = 2.915, *p* = 0.005), whereas BMR did not significantly change with body mass (*T*_69_ = 1.049, *p* = 0.298). Time of day, dietary AOX, and sex were not significantly related to metabolic rates (Supporting Information Appendix B: Table B‐1). BMR and PMR were not correlated with each other (*T*_72_ = 0.501, *p* = 0.618).

### 3.2 Exercise performance

**Figure 2:**
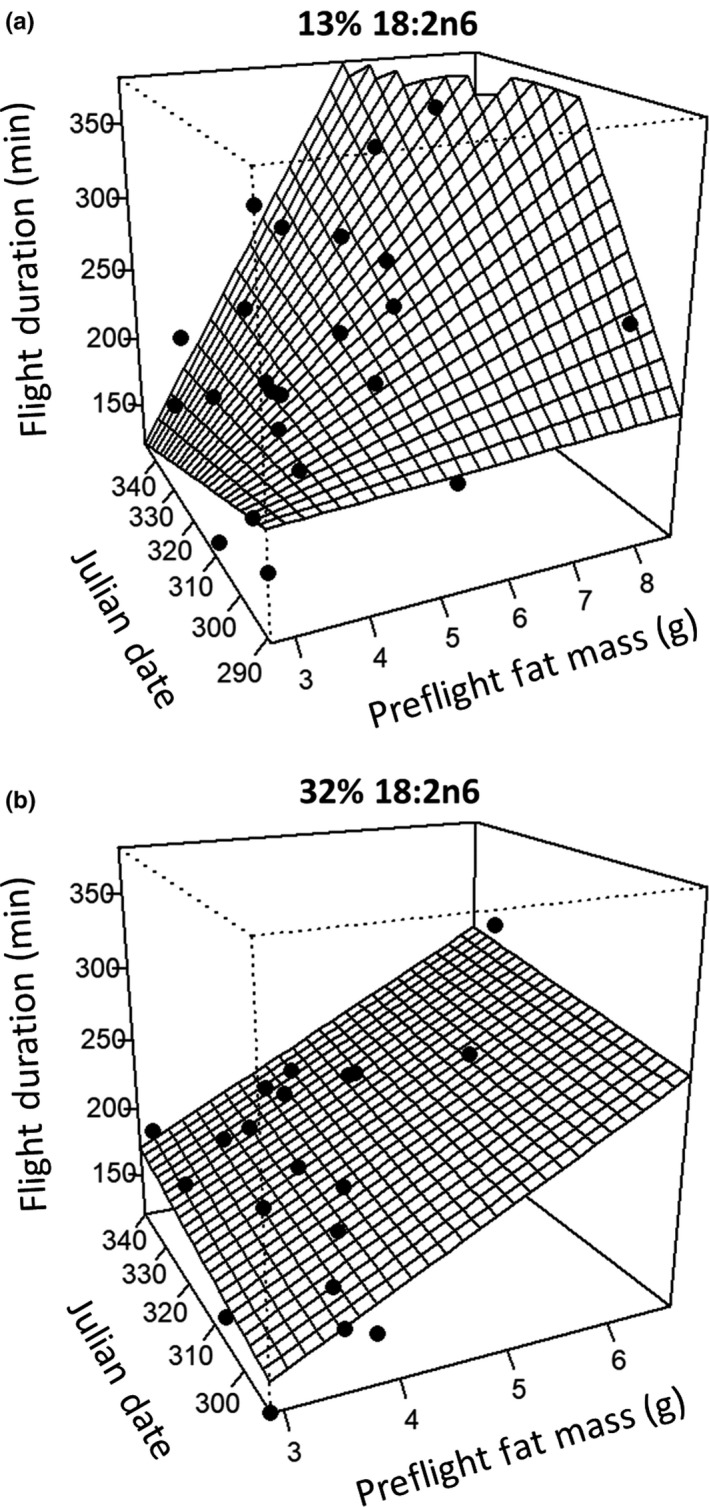
Flight durations of starlings fed low (13%, a) or high (32%, b) concentrations of 18:2n6 during a voluntary flight of >120 min at the culmination of the 15‐day flight‐training regimen. Preflight fat mass (±*SE*) was measured using a QMR

Preflight fat mass did not significantly change over time (*T*_39_ = −0.095, *p* = 0.925) or differ between diet groups (*T*_39_ = −1.067, *p* = 0.292; Table 2). There was a trend for preflight lean mass to decrease over the course of the experiment (*T*_39_ = −2.019, *p* = 0.051), but lean mass did not differ between diet groups (*T*_39_ = −0.588, *p* = 0.560; Table 2). As with the metabolic rates, we found significant relationships between dietary FA composition, date, and our measures of exercise performance (Supporting Information Appendix B: Table B‐2). For flight duration, there was a significant three‐way interaction between dietary FA composition, date, and preflight fat mass (*T*_32_ = −2.084, *p* = 0.045): flight duration in the 32% group was stable over successive cohorts and increased with preflight fat mass, whereas flight duration in the 13% group increased with the product of date and preflight fat mass (Figure 2) resulting in birds with higher fat loads increasing their flight duration over successive cohorts.

**Table 2:** Preflight body masses, lean masses, and fat masses for trained birds on day 15 of flight training

Diet group	Preflight body mass (g)	Preflight lean mass (g)	Preflight fat mass (g)
13%L	78.27 ± 1.3	62.84 ± 1.03	4.3 ± 0.49
13%H	75.09 ± 0.95	60.54 ± 0.94	3.89 ± 0.31
32%L	76.78 ± 1.1	62.65 ± 1.03	3.24 ± 0.28
32%H	74.69 ± 1.54	60.36 ± 1.35	3.33 ± 0.36

Note

Values are means ± SE for each diet group.

**Figure 3:**
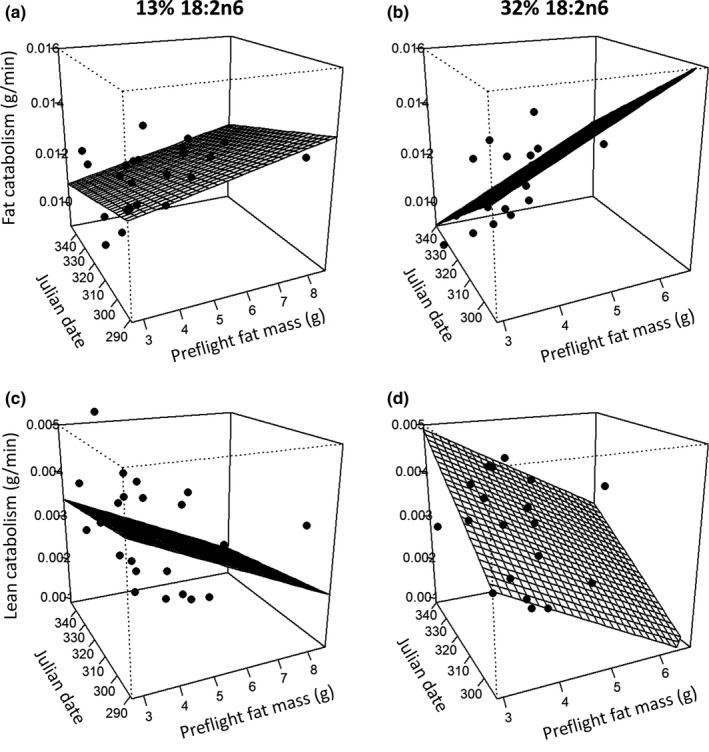
Rates of fat and lean catabolism during flight for Starlings fed low (13%, a and c) or high (32%, b and d) concentrations of 18:2n6. Preflight fat mass was measured using a QMR. Rates of fat and lean catabolism were calculated by dividing the fat or lean mass lost during a long flight of >120 min by the duration of the flight

The rate of fat catabolism was subject to significant interactions between dietary FA composition and preflight fat mass (*T*_34_ = 2.885, *p* = 0.006) and between dietary FA composition and date (*T*_34_ = −3.812, *p* < 0.001): Fat catabolism in the 32% group decreased over successive cohorts and increased with fat mass, but was relatively stable across time and fat masses in the 13% group (Figure 3a,b). The rate of lean catabolism decreased for all birds as fat mass increased (*T*_35_ = −2.187, *p* = 0.036) and, similar to fat catabolism, was influenced by an interaction between dietary FA composition and date (*T*_35_ = 2.289, *p* = 0.028): Lean catabolism increased over time in the 32% group but was relatively stable over successive cohorts in the 13% group (Figure 3c,d).

**Figure 4:**
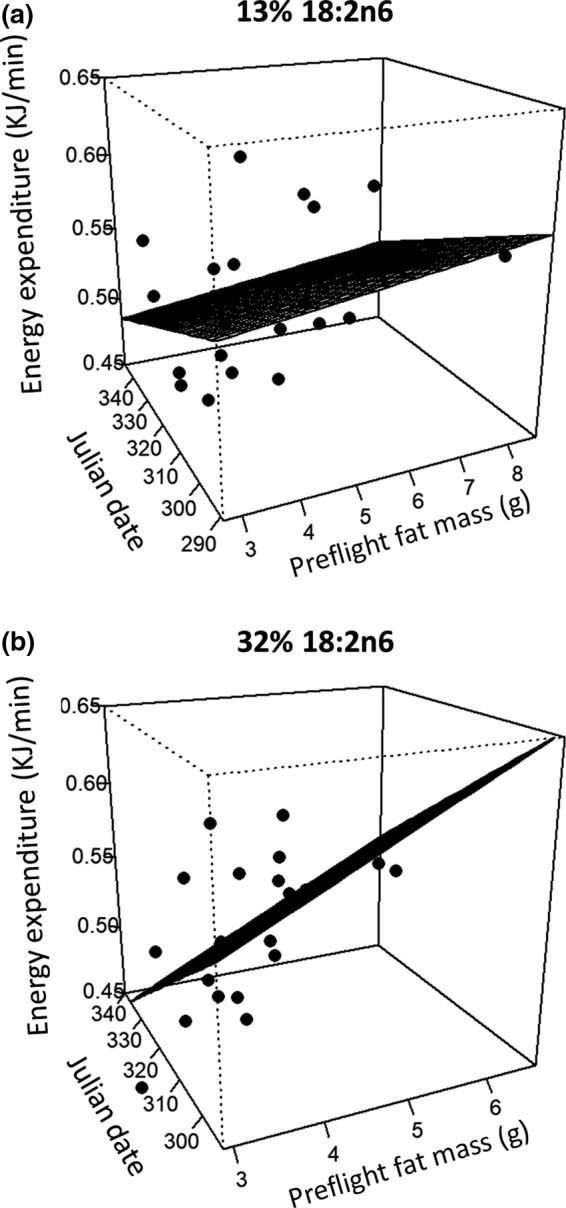
Rates of energy expenditure during flight for starlings fed low (13%, a) or high (32%, b) concentrations of 18:2n6. Preflight fat mass was measured with a QMR. Energy expenditure (kJ/min) was calculated by multiplying the fat and lean mass lost over the course of a >120‐min flight by their respective energy densities, summing them, and dividing by the duration of the flight

Energy expenditure exhibited a similar pattern to fat catabolism with significant interactions between dietary FA composition and preflight fat mass (*T*_34_ = 2.386, *p* = 0.023) and between dietary FA composition and date (*T*_34_ = −2.458, *p* = 0.019): The rate of energy expenditure increased with fat mass and decreased over successive cohorts in the 32% group but was relatively stable across fat masses and cohorts in the 13% group (Figure 4). Body mass was a significant covariate in models of energy expenditure (*T*_34_ = 2.950, *p* = 0.006) and fat catabolism (*T*_34_ = 3.730, *p* < 0.001), but not flight duration (*T*_32_ = 0.694, *p* = 0.493) or lean catabolism (*T*_35_ = −1.01, *p* = 0.320). Neither sex nor dietary AOX were significantly related to exercise performance (Supporting Information Appendix B: Table B‐2). Fat catabolism was positively correlated with energy expenditure (*ρ* = 0.932, *T*_41_ = 16.51, *p* < 0.001) and lean catabolism was negatively correlated with flight duration (*ρ* = −0.507, *T*_41_ = −3.764, *p* = 0.008) and fat catabolism (*ρ* = −0.342, *T*_41_ = −2.329, *p* = 0.025), but none of the measures of exercise performance were correlated with BMR or PMR (all *p* > 0.336).

### 3.3 Tissue fatty acid composition

**Figure 5:**
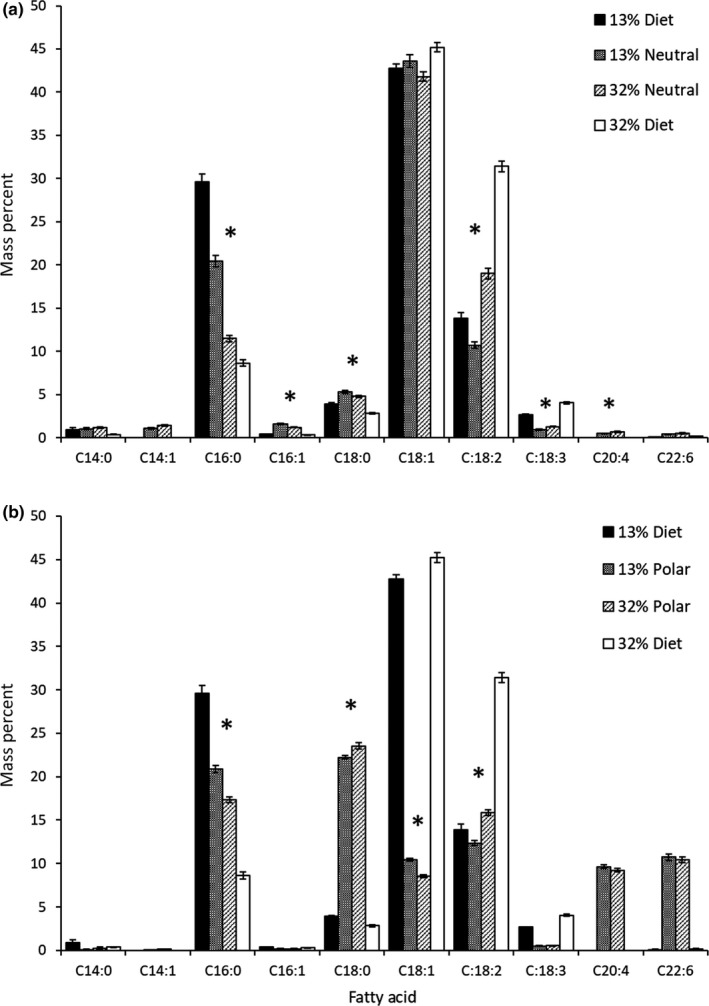
Fatty acid composition of experimental diets and pectoralis muscle lipid droplets (Neutral, a) and membranes (Polar, b) in starlings. Composition is presented for the ten most concentrated fatty acids as the percent of total fatty acids by mass. Asterisks denote significant differences between diet groups (*p* < 0.05)

As expected given the experimental diets, pectoralis muscle fatty acid profiles of both lipid droplets (Neutral fraction; $T_{5,72}^{2}$ = 46.002, *p* < 0.0001) and membranes (Polar fraction; $T_{6,76}^{2}$ = 38.177, *p* < 0.0001) significantly differed between diet FA groups (Figure 5, Supporting Information Appendix C: Table C‐1). Lipid droplets were primarily composed of palmitic acid (16:0), oleic acid (18:1n9), and 18:2n6. Concentrations of 16:0 were higher in the 13% 18:2n6 group (*T*_72_ = 2.396, *p* = 0.0192) whereas 18:2n6 was higher in the 32% 18:2n6 group (*T*_73_ = 11.643, *p* < 0.0001), but both had higher concentrations when birds were trained (16:0, *T*_72_ = −4.854, *p* < 0.0001; 18:2n8, *T*_73_ = −2.366, *p* = 0.0206; Supporting Information Appendix C: Figure C‐1). There was also an increase in 16:0 over successive cohorts that was specific to the 13% 18:2n6 group (*T*_72_ = −3.202, *p* = 0.002; Supporting Information Appendix C: Figure C‐1). In contrast, membranes contained appreciable concentrations of 16:0, 18:0, 18:1n9, 18:2n6, arachidonic acid (20:4n6), and docosahexaenoic acid (22:6n3). Concentrations of both 16:0 (*T*_78_ = −6.756, *p* < 0.0001) and 18:1n9 (*T*_78_ = −7.7471, *p* < 0.0001) were higher in the 13% 18:2n6 group, whereas 18:0 (*T*_78_ = 3.530, *p* = 0.0007) and 18:2n6 (*T*_78_ = 9.075, *p* < 0.0001) were higher in the 32% 18:2n6 group. As with lipid droplets, 16:0 (*T*_78_ = −2.335, *p* = 0.0221) and 18:2n6 (*T*_78_ = −3.106, *p* = 0.0026) were more concentrated when birds were trained, but 18:0 (*T*_78_ = 2.170, *p* = 0.033) and 22:6n3 (*T*_78_ = 5.095, *p* < 0.0001) were higher when birds were untrained (Supporting Information Appendix C: Figure C‐2). Meanwhile, 18:1n9 had lower concentrations when birds were supplemented with anthocyanins (*T*_78_ = 2.153, *p* = 0.0344). Fatty acid concentrations were stable over time in membranes except for subtle decreases in 18:0 (*T*_78_ = −2.470, *p* = 0.0157) and increases in 18:2n6 (*T*_78_ = 2.516, *p* = 0.0139). The only correlations between fatty acid composition and whole‐animal performance both involved lipid droplet 16:0, which was positively related to flight duration (*ρ* = 0.407, *T*_38_ = 2.748, *p* = 0.0091) and negatively related to lean catabolism (*ρ* = −0.377, *T*_38_ = −2.509, *p* = 0.0165). However, both of these correlations were driven by differences between diet groups.

### 3.4 Plasma metabolites

**Figure 6:**
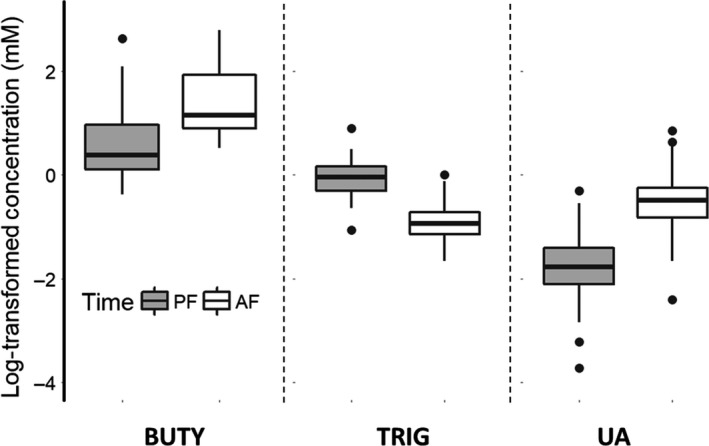
Log‐transformed concentrations of β‐hydroxybutyrate (BUTY), triglycerides (TRIG), and uric acid (UA) in the plasma of starlings before (PF) and after (AF) the culminating flight of a 15‐day flight‐training regimen. There were significant increases in BUTY and UA and decreases in TRIG between pre‐ and postflight indicating significant catabolism of fat and protein during the longest‐duration flights

Log‐transformed plasma β‐hydroxybutyrate concentrations were significantly elevated in postflight samples relative to preflight samples (Figure 6; *T*_49_ = 10.475, *p* < 0.001). Conversely, log‐transformed triglyceride concentrations were significantly depleted in postflight samples (Figure 6; *T*_95_ = −12.405, *p* < 0.001). Log‐transformed uric acid concentrations were elevated in postflight samples (Figure 6, *T*_46.55_ = 11.326, *p* < 0.001) and also increased over the course of the study (*T*_45.41_ = 2.134, *p* = 0.038). Although the variance associated with individual was only significant for β‐hydroxybutyrate (BUTY: $X_{1}^{2}$ = 25.05, *p* < 0.0001; TRIG: $X_{1}^{2}$ = 0.01, *p* = 0.99; UA: $X_{1}^{2}$ = 2.74, *p* < 0.098), removal of the random effect from the other models did not qualitatively change these results. Postflight uric acid concentrations were positively related to flight duration (*ρ* = 0.327, *T*_48_ = 2.400, *p* = 0.020). Dietary FA and AOX composition had no effect on any metabolite concentrations.

## 4 DISCUSSION

### 4.1 Dietary fatty acids and whole‐animals performance

In contrast to previous studies with similar, but not identical, diet manipulations (McWilliams & Pierce, 2006; Pierce & McWilliams, 2005; Price & Guglielmo, 2009), our diet manipulation did not produce static differences in performance between diet groups. Instead, we found similar patterns of diet‐specific change over the course of the experiment in most of our measures, with birds fed the 32% 18:2n6 diet experiencing decreases in BMR, PMR, rate of fat catabolism, and rate of energy expenditure over time and birds fed the 13% 18:2n6 diet increasing in those measures over time. The two exceptions were the rate of lean catabolism, which exhibited a mirrored pattern of decrease in the 13% 18:2n6 group and increase in the 32% 18:2n8 group, and flight duration, which did not change over time in the 32% 18:2n6 group but increased with both time and fat load in the 13% 18:2n6 group. Additionally, flight duration, fat catabolism, and energy expenditure all displayed diet‐specific relationships with preflight fat load. Thus, a complete explanation of these results will both link dietary FA composition with performance and describe how this relationship changes with time and fat load.

One explanation is that PUFAs generally have higher mobilization and diffusion rates than MUFAs or SFAs of similar size (Price, Krokfors, & Guglielmo, 2008; Raclot, 2003). Thus, fat stores with higher concentrations of PUFAs could be transported to the mitochondria at higher rates, resulting in higher metabolic rates and rates of fat catabolism (Price, Staples, Milligan, & Guglielmo, 2011). This is consistent with the higher concentrations of 18:2n6 in muscle lipid droplets, basal and peak metabolic rates, and rates of fat catabolism and energy expenditure in the 32% 18:2n6 group early in the experiment. Similarly, higher concentrations of 18:2n6 could result in greater total amounts of fat mobilized in the course of a flight, explaining the diet‐specific relationship between fat catabolism and fat load. However, the concentration of 18:2n6 in lipid droplets remained stable throughout the experiment and so cannot explain the diet‐specific changes in performance over time. Lipid droplet 18:2n6 concentration was also not correlated with any measure of performance, as we would expect whether it was directly responsible for the observed effects of diet.

Alternately, several studies have found correlations between concentrations of PUFA in cell and organelle membranes and enzyme activity in mammalian and avian muscle (Giroud et al., 2013; Maillet & Weber, 2007; Turner, Haga, Else, & Hulbert, 2006). Higher concentrations of 18:2n6 in the muscle membranes of birds fed the 32% 18:2n6 diet and correspondingly higher activity of oxidative and transport enzymes could thus explain the higher BMR, PMR, rate of fat catabolism, and rate of energy expenditure observed in that group early in the experiment, although once again these concentrations were stable throughout the experiment whereas performance changed. It is also unclear why changes in membrane and membrane‐bound enzyme properties would affect birds differently depending on the fat load carried during flight. Finally, membrane 18:2n6 concentration was not related to any measure of performance, as would be expected whether membrane properties were directly responsible for the observed effects of diet.

Fatty acids, especially PUFAs, are also known to have a number of signaling functions, acting as ligands for peroxisome proliferator‐activated receptors (PPARs) and precursor molecules for eicosanoid hormones (Corder et al., 2016; Nagahuedi, Popesku, Trudeau, & Weber, 2009; Sampath & Ntambi, 2004). PPARs, in particular, are involved in the regulation of fat oxidation (Demoranville et al., 2019; Weber, 2011; Wolfrum & Spener, 2000), and so upregulation of these pathways after exposure to higher dietary concentrations of 18:2n6 could result in greater oxidative capacities for birds in the 32% 18:2n6 group, again matching the elevated BMR, PMR, rate of fat catabolism, and rate of energy expenditure in this group early in the experiment. Concentration‐dependent negative feedback to 18:2n6 exposure could then explain diet‐specific changes in metabolism over successive cohorts (Fujimori, 2012; Inoue, Tanabe, & Umesono, 2000). It is also plausible that diet‐specific changes in metabolism over cohorts and fat loads can be explained by integrating innate circannual‐ and body condition‐based influences on the regulation of oxidative capacity (Batista‐Pinto, Rocha, Castro, Rodrigues, & Lobo‐da‐cunha, 2009; Corder et al., 2016; McFarlan et al., 2009; Zhang, King, Harmon, Eyster, & Swanson, 2015). The changes in metabolism we observed over the course of this experiment occurred in the absence of external temperature and photoperiod cues. However, an innately triggered shift to a migration or overwintering phenotype would likely involve changes in the regulation of metabolism which, when combined with regulation of metabolism by diet, could produce the interacting patterns that we observed. Finally, signaling effects are indirect and so do not require correlations between tissue PUFA composition and performance.

Of the three mechanisms discussed here, none are clearly rejected based on the results of this study. However, the fuel hypothesis does not adequately explain changes over time, the membrane hypothesis does not adequately explain changes over fat loads, and both predict correlations between tissue fatty acid composition and performance that were absent. On balance, it appears that the signaling hypothesis is best supported and future studies should seek to test the mechanistic links (e.g., PPAR density, oxidative enzyme density, and activity) between diet and whole‐animal performance that this hypothesis predicts.

### 4.2 Dietary antioxidants and whole‐animal performance

The accumulation of oxidative damage from the byproducts of aerobic metabolism is expected to reduce whole‐animal performance either directly, through reduced cellular and organismal function (Eikenaar, Isaksson, & Hegemann, 2018), or indirectly, as resources are directed to endogenous antioxidant responses rather than oxidative capacity (Cooper‐Mullin & McWilliams, 2016; Larcombe, Herborn, Alexander, & Arnold, 2017). The potential for reduced performance is particularly high for individuals with high tissue PUFA concentrations, due to elevated risk of peroxidation (Hulbert, 2010; Skrip & McWilliams, 2016). Dietary antioxidants have been proposed to relieve these tradeoffs (Catoni, Schaefer, et al., 2008; Larcombe et al., 2008; Skrip et al., 2016), by either enhancing preparation for oxidative stress or speeding recovery and preventing the accumulation of damage (Beaulieu & Schaefer, 2013). Faster recovery and reduced damage to metabolically active tissues could result in greater oxidative capacity and endurance, manifested in higher peak metabolic rates or longer flight durations.

In contrast to these predictions, we found no influence of dietary antioxidants on any of our measures of whole‐animal performance. This negative result could be the product of a low accumulation of oxidative damage from flight training and/or the high‐PUFA diet as was found in a previous wind tunnel study (Dick & Guglielmo, 2019b), although the variance in PUFA content of the diets in that study was much smaller. It is also possible that the hydrophilic antioxidants used to supplement the diets simply did not remain in circulation at high enough concentrations to prophylactically counter oxidative damage and failed to support recovery from oxidative damage (Beaulieu & Schaefer, 2013; Cooper‐Mullin & McWilliams, 2016). One other speculative explanation is that endogenous antioxidants such as uric acid (Alan & McWilliams, 2013; Machin, Simoyi, Blemings, & Klandorf, 2004) could be preferentially used to counter high RS production. An emphasis on uric acid production would help explain increasing rates of lean tissue catabolism over the course of the experiment in the 32% 18:2n6 diet group, although circulating uric acid concentrations did not themselves differ between diet groups either before or after flight. Future measurements of antioxidant capacity and oxidative damage may better distinguish between these possibilities.

### 4.3 Interrelations between basal, peak, and sustained metabolism

As with a number of previous studies (Barceló, Love, & Vézina, 2017; McKechnie & Swanson, 2010; Swanson, Thomas, Liknes, & Cooper, 2012), we found no correlation between BMR and PMR or between either and any of our measures of sustained metabolism in the wind tunnel. This discontinuity suggests that basal, sustained, and peak metabolism are regulated differently from one another, albeit with a shared, underlying link to diet. Variation in BMR can result from variation in the relative sizes and metabolic activities of a wide range of tissues (Konarzewski & Kjiazek, 2013; Vézina & Williams, 2005), and so the influence of diet on BMR in this study may have resulted from variation in the metabolic activity of the liver and other tissues that are always in use. In contrast, peak metabolism during physical activity is much more closely related to the capabilities of skeletal muscle and fuel transport (Wiersma, Chappell, & Williams, 2007; Zhang, Eyster, Liu, & Swanson, 2015) and for birds is likely limited by the abundance of oxidative enzymes and lipid transporters in flight muscle (Barceló et al., 2017; Jenni & Jenni‐Eiermann, 1998; Vézina & Williams, 2005). The metabolic activity of both skeletal muscle and visceral organs can be influenced by fatty acid‐based signaling pathways (Batista‐Pinto et al., 2009; Corder et al., 2016; Sampath & Ntambi, 2004).

Sustained metabolism during physical activity is similarly related to the characteristics of skeletal muscle and fuel supply, but is regulated below maximal levels (Hammond & Diamond, 1997; Wiersma et al., 2007). This regulation is perhaps most readily modulated at either the initial release of triacylglycerols by lipases in adipose tissue or by negative feedback of transport and oxidation in the mitochondria (Hammond & Diamond, 1997; McClelland, 2004; Weber, 2011). Both of these steps can be regulated by fatty acid‐based signaling pathways (Corder et al., 2016; Kim & Lee, 2010) and could thus result in diet‐dependent regulation of sustained fat catabolism during physical activity. Upregulation of fat catabolism could, in turn, downregulates lean catabolism (Gerson & Guglielmo, 2013; Jenni & Jenni‐Eiermann, 1998; Yeo et al., 2008), leading to the inverse relationship observed between the two.

### 4.4 Diet, whole‐animal performance, and songbird migratory ecology

Migration is an energy‐intensive life history stage, and failure to meet these energetic challenges can lead to high rates of mortality during migratory periods (Dingle, 2014; Rockwell et al., 2017; Ward et al., 2018; Wikelski et al., 2003). Over the course of a migration, relatively small differences in metabolism could accumulate and lead to substantial differences in the energetic requirements and success of migrants. As with previous studies (McWilliams & Pierce, 2006; Pierce et al., 2005; Price & Guglielmo, 2009), our results demonstrate that dietary fatty acids are capable of producing such differences in metabolism, with dietary 18:2n6 apparently mediating many of these effects. However, our finding of variable dietary effects over time means that the benefits of a given diet are likely to be dependent on context. In the shorter term, a diet high in 18:2n6 led to less efficient energy use during flight, but also lower rates of lean catabolism which could help preserve muscle tissue during flight and lead to faster recovery postflight. Similarly, if the elevated BMR of the 32% 18:2n6 group early in the experiment was the result of larger or more metabolically active digestive organs, it may be indicative of an increased ability to gain mass in preparation for migratory flights. In contrast, the decreased BMR, more energy efficient flight, and higher protein catabolism resulting from prolonged exposure to a high 18:2n6 diet could provide the energy savings necessary to complete migratory flights under energetic constraints. For example, over the course of a three‐hour flight an average‐sized, late‐season starling carrying 3 g of fat and fed a high 18:2n6 diet would spend approximately 77 kJ of energy whereas a similarly sized and laden bird fed a low 18:2n6 diet would spend approximately 99 kJ. This represents a roughly 17% increase in energy used that would require the consumption of an additional 2–6 g of fruits like those available during migration in southern New England (Smith et al., 2007). The time course of these effects could potentially be aligned with the migratory period to maximize benefits: Fattening and maintaining muscle mass would be most important early in migration, while efficiency could be most important for ensuring survival later in migration (Smith & McWilliams, 2014; Ward et al., 2018). It is important, however, to note that it is unclear how efficiency during wind tunnel flight translates into behavior and efficiency in the wild when birds are less constrained and can choose their speed and flight space (Engel et al., 2006; Jenni‐Eiermann et al., 2002; Rothe, Biesel, & Nachtigall, 1987). Future studies may find it profitable to investigate the effects of dietary fatty acids without these constraints. Fatty acids in the n6 family, including 18:2n6, are found more commonly in terrestrial ecosystems than aquatic systems (Klasing, 1998; Martinez del Rio & McWilliams, 2016) and are found in high concentrations in many of the fruits that migratory songbirds consume during fall migration (Boyles, 2011; Smith et al., 2007; Zurovchak, 1997). Similar variation in 18:2n6 content also exists among insects that differ in diets and the ability to synthesize n6 PUFAs (Blomquist, Borgeson, & Vundla, 1991; Stanley‐Samuelson, Jurenka, Cripps, Blomquist, & de Renobales, 1988). Thus, songbirds may be able to selectively manipulate their dietary intake of 18:2n6 and closely match their metabolism to the needs of their current life history stage.

## 5 CONCLUSION

In this study, we directly tested the effect of dietary 18:2n6 on whole‐animal performance and demonstrated that it influences the basal, peak, and sustained metabolism of European starlings. However, in contrast to previous studies conducted over shorter periods, the influence of diet changed over the course of the experiment with the 32% 18:2n6 diet associated with higher metabolism early in the experiment and lower metabolism late in the experiment. In contrast, the 13% 18:2n6 diet was associated with metabolic rates that were more stable or demonstrated an increasing trend over the course of the experiment. We propose that these patterns are best explained by signaling properties of 18:2n6 and its derivatives, which led to an upregulation of fat metabolism early in the experiment and then either experienced negative feedback with prolonged exposure or integrated innate circannual changes in metabolism in a diet‐dependent manner. The diverse and indirect signaling effects of 18:2n6 also help to explain the apparent independence of its influence on basal, peak, and sustained metabolism. Contrary to our expectations, we found no influence of dietary antioxidants on whole‐animal metabolism, indicating either a lack of substantial oxidative stress or an inability to effectively route hydrophilic antioxidants to the sites of damage prophylactically or palliatively. This study again highlights the importance of dietary fatty acids for songbird performance and suggests that they could influence songbird ecology especially given their availability in terrestrial food sources.

## CONFLICT OF INTEREST

None declared.

## AUTHOR CONTRIBUTIONS

SRM and BJP conceived of and designed the experiment. WAC and KJD refined the methodology and conducted the experiment. WAC analyzed the data, and WAC and SRM primarily wrote the manuscript; all other authors provided editorial advice to the manuscript.

## Data Availability

The datasets used in this manuscript are archived in the Dryad data repository and available at the following https://doi.org/10.5061/dryad.bg79cnp7j.
