# Supplementary figures and images for: The effects of dietary linoleic acid and hydrophilic antioxidants on basal, peak, and sustained metabolism in flight‐trained European starlings
Source: Ecol Evol. 2020 Jan 18;10(3):1552–66. doi: 10.1002/ece3.6010 (PMC7029098; doi:10.1002/ece3.6010)

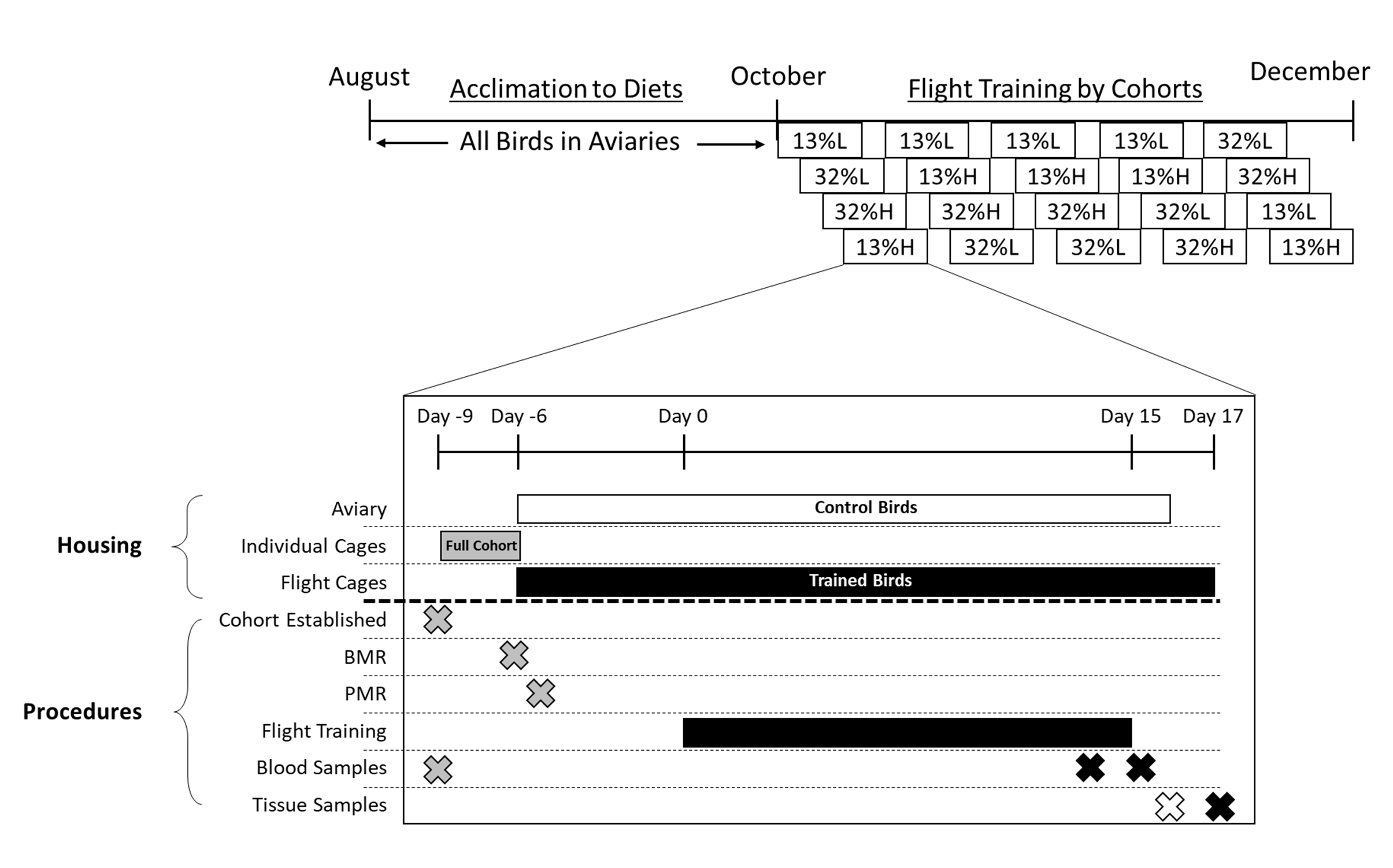

Supplement: Supplementary file 1 [file ECE3-10-1552-s001.tif]

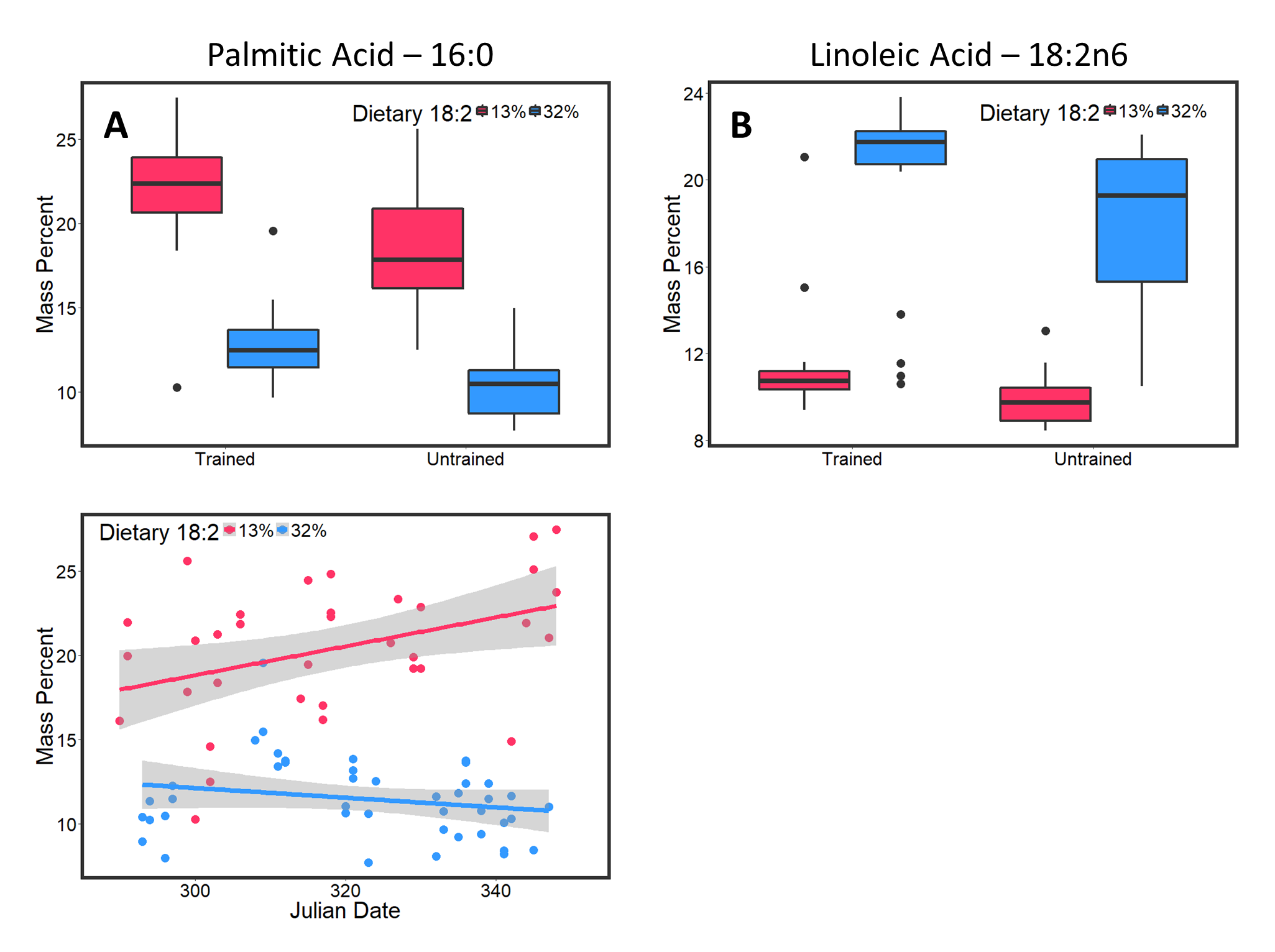

Supplement: Supplementary file 3 [file ECE3-10-1552-s003.tif]

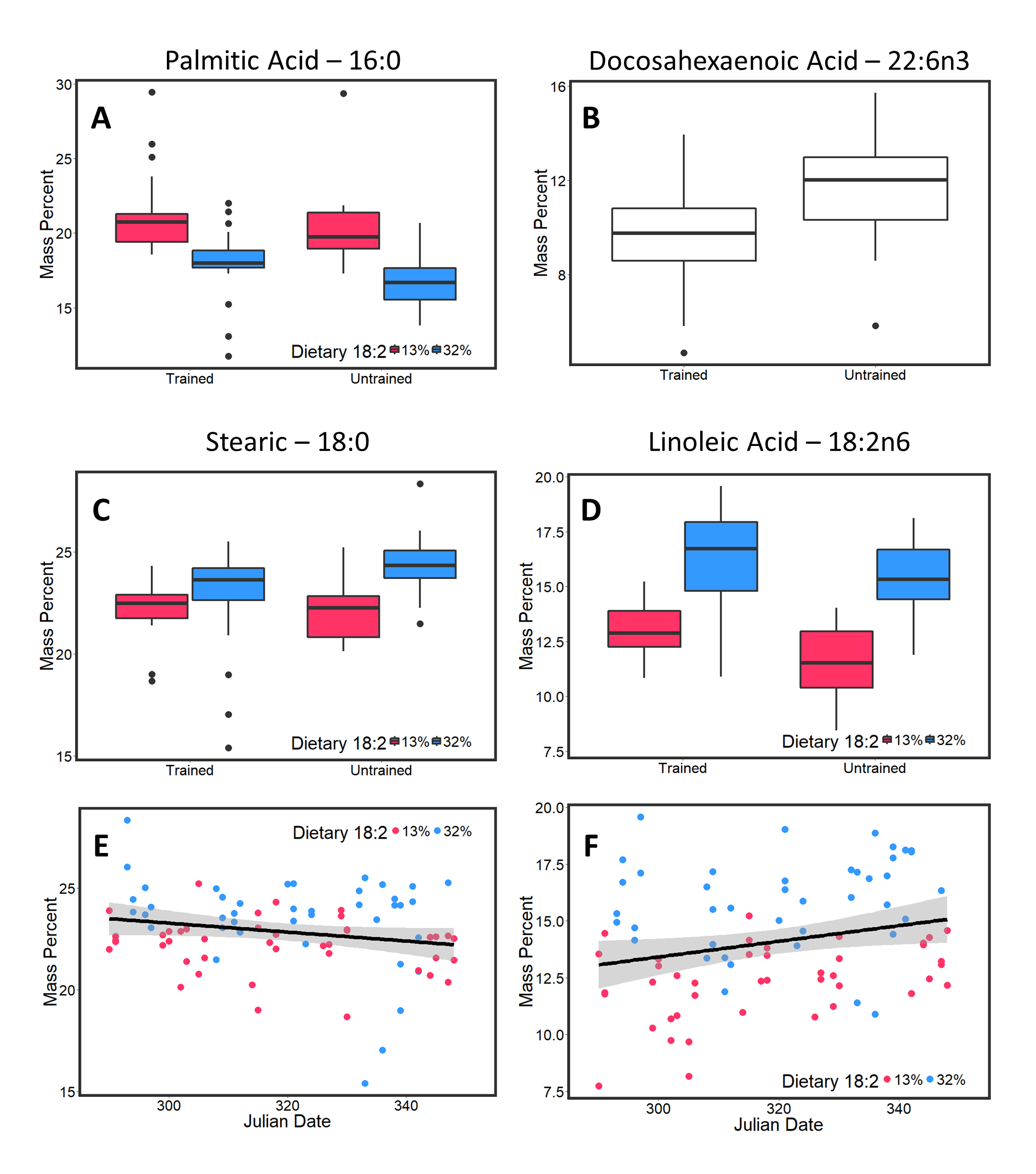

Supplement: Supplementary file 4 [file ECE3-10-1552-s004.tif]
