# Supplementary material for: The effects of dietary linoleic acid and hydrophilic antioxidants on basal, peak, and sustained metabolism in flight‐trained European starlings
Source: Ecol Evol. 2020 Jan 18;10(3):1552–66. doi: 10.1002/ece3.6010 (PMC7029098; doi:10.1002/ece3.6010)
Supplement: Supplementary file 2 [file ECE3-10-1552-s002.docx]

*Appendix B: Final Models*

Table B-1. Parameter estimates and Wald statistics for final models of BMR and PMR. The diet with high 18:2n6 and high antioxidants is the reference group for both models. Colons indicate interactions between model effects and significant p-values are in bold.

| Coefficients: | Estimate | SE | *t* - value | *P* |
| --- | --- | --- | --- | --- |
| BMR |  |  |  |  |
| Intercept | 162.414 | 69.268 | 2.345 | **0.0219** |
| Body Mass | 0.614 | 0.585 | 1.049 | 0.2980 |
| Date | -0.261 | 0.169 | -1.542 | 0.1277 |
| Dietary FA | -218.381 | 67.131 | -3.253 | **0.0018** |
| Sex | 4.745 | 4.929 | 0.963 | 0.3391 |
| Dietary AOX | 4.848 | 3.883 | 1.249 | 0.2160 |
| Date : Dietary FA | 0.724 | 0.224 | 3.228 | **0.0019** |
| *R*^2^ = 0.194, *df* = 69 |  |  |  |  |
|  |  |  |  |  |
| PMR |  |  |  |  |
| Intercept | 1945.869 | 829.083 | 2.347 | **0.0218** |
| Body Mass | 19.344 | 6.636 | 2.915 | **0.0048** |
| Time | -930.181 | 636.812 | -1.461 | 0.1487 |
| Date | -4.190 | 1.957 | -2.141 | **0.0358** |
| Dietary FA | -1625.900 | 749.705 | -2.169 | **0.0336** |
| Sex | -59.024 | 55.870 | -1.056 | 0.2945 |
| Dietary AOX | 26.253 | 43.240 | 0.607 | 0.5458 |
| Date : Dietary FA | 5.308 | 2.505 | 2.118 | **0.0378** |
| *R*^2^ = 0.196, *df* = 68 |  |  |  |  |

Table B-2. Parameter estimates and Wald statistics for final models of flight duration, fat catabolism, lean catabolism, and energy expenditure. The low 18:2n6, high antioxidant diet is the reference group for all models. Significant p-values are in bold.

| Coefficients: | Estimate | SE | *t* - value | *P* |
| --- | --- | --- | --- | --- |
| Flight Duration |  |  |  |  |
| Intercept | 1486.355 | 456.228 | 3.258 | **0.0027** |
| Body Mass | 1.159 | 1.669 | 0.694 | 0.4925 |
| Pre-Flight Fat Mass | -358.326 | 101.566 | -3.528 | **0.0013** |
| Date | -4.714 | 1.441 | -3.272 | **0.0026** |
| Dietary FA | -2147.992 | 970.870 | -2.212 | **0.0342** |
| Sex | -15.639 | 16.551 | -0.945 | 0.3518 |
| Dietary AOX | -10.929 | 10.891 | -1.004 | 0.3231 |
| Pre-Flight Fat Mass: Date | 1.242 | 0.325 | 3.820 | **0.0006** |
| Pre-Flight Fat Mass: Dietary FA | 521.563 | 256.455 | 2.034 | 0.0503 |
| Date : Dietary FA | 6.739 | 3.004 | 2.244 | **0.0319** |
| Pre-Flight Fat Mass : Date : Dietary FA | -1.643 | 0.788 | -2.084 | **0.0452** |
| *R*^2^ = 0.747, *df* = 32 |  |  |  |  |
|  |  |  |  |  |
| Fat Catabolism |  |  |  |  |
| Intercept | 4.220e-03 | 4.970e-03 | 0.849 | 0.4018 |
| Body Mass | 1.613e-04 | 4.326e-05 | 3.730 | **0.0007** |
| Pre-Flight Fat Mass | 8.695e-05 | 1.376e-04 | 0.632 | 0.5318 |
| Date | -1.441e-05 | 1.125e-05 | -1.280 | 0.2090 |
| Dietary FA | 1.751e-02 | 5.265e-03 | 3.326 | **0.0021** |
| Sex | -6.013e-04 | 4.272e-04 | -1.408 | 0.1684 |
| Dietary AOX | 1.127e-04 | 2.798e-04 | 0.403 | 0.6896 |
| Pre-Flight Fat Mass: Dietary FA | 7.506e-04 | 2.601e-04 | 2.885 | **0.0067** |
| Date : Dietary FA | -6.480e-05 | 1.700e-05 | -3.812 | **0.0006** |
| *R*^2^ = 0.670, *df* = 34 |  |  |  |  |
|  |  |  |  |  |
| Lean Catabolism |  |  |  |  |
| Intercept | 7.088e-03 | 5.423e-03 | 1.307 | 0.1998 |
| Body Mass | -4.764e-05 | 4.718e-05 | -1.010 | 0.3195 |
| Pre-Flight Fat Mass | -2.931e-04 | 1.340e-04 | -2.187 | **0.0355** |
| Date | 4.396e-07 | 1.225e-05 | 0.036 | 0.9716 |
| Dietary FA | -1.290e-02 | 5.741e-03 | -2.248 | **0.0310** |
| Sex | 7.151e-04 | 4.630e-04 | 1.545 | 0.1314 |
| Dietary AOX | 5.645e-04 | 3.055e-04 | 1.848 | 0.0731 |
| Date : Dietary FA | 4.118e-05 | 1.799e-05 | 2.289 | **0.0282** |
| *R*^2^ = 0.379, *df = 35* |  |  |  |  |
|  |  |  |  |  |
| Energy Expenditure |  |  |  |  |
| Intercept | 0.2975 | 0.2121 | 1.403 | 0.1697 |
| Body Mass | 0.0054 | 0.0018 | 2.950 | **0.0057** |
| Pre-Flight Fat Mass | -0.0010 | 0.0059 | -0.163 | 0.8717 |
| Date | -0.0006 | 0.0005 | -1.194 | 0.2407 |
| Dietary FA | 0.4607 | 0.2246 | 2.051 | **0.0481** |
| Sex | -0.0117 | 0.0182 | -0.643 | 0.5243 |
| Dietary AOX | 0.0142 | 0.0119 | 1.192 | 0.2415 |
| Pre-Flight Fat Mass: Dietary FA | 0.0265 | 0.0111 | 2.386 | **0.0227** |
| Date : Dietary FA | -0.0018 | 0.0007 | -2.458 | **0.0192** |
| *R*^2^ = 0.563, *df* = 34 |  |  |  |  |
