# Supplementary material for: The effects of dietary linoleic acid and hydrophilic antioxidants on basal, peak, and sustained metabolism in flight‐trained European starlings
Source: Ecol Evol. 2020 Jan 18;10(3):1552–66. doi: 10.1002/ece3.6010 (PMC7029098; doi:10.1002/ece3.6010)
Supplement: Supplementary file 5 [file ECE3-10-1552-s005.docx]

*Appendix C: Effects of time, diet, and training on tissue fatty acid composition*

Table C-1. Fatty acid composition of pectoralis muscle lipid droplets (Neutral lipid fraction) and membranes (Polar lipid fraction) of starlings fed diets differing in 18:2n6 concentration. Composition of the ten most concentrated fatty acids is presented as mean percent of total fatty acids by mass ± standard deviation.

| Lipid Fraction | Diet | Fatty Acid | Cohort 1 | Cohort 2 | Cohort 3 | Cohort 4 | Cohort 5 |
| --- | --- | --- | --- | --- | --- | --- | --- |
|  |  |  | 54 - 64 Days on Diet | 66 -76 Days on Diet | 78 - 88 Days on Diet | 90 - 100 Days on Diet | 102-112 Days on Diet |
| Neutral | 13% | C14:0 | 1.09±0.19 | 1.14±0.33 | 1.13±0.14 | 0.75±0.08 | 0.96±0.2 |
|  |  | C15:1 | 1.09±0.19 | 1.09±0.24 | 1.31±0.13 | 0.97±0.06 | 1.04±0.21 |
|  |  | C16:0 | 19.06±1.46 | 18.5±1.38 | 20.53±1.15 | 20.88±0.61 | 24.89±0.87 |
|  |  | C16:1 | 1.53±0.14 | 1.6±0.24 | 1.64±0.1 | 1.7±0.14 | 1.35±0.27 |
|  |  | C18:0 | 5.11±0.32 | 5.33±0.49 | 4.9±0.23 | 5.37±0.08 | 6.13±0.29 |
|  |  | C18:1 | 43.3±1.58 | 42.95±2 | 42.99±0.86 | 46.51±1.21 | 42.39±0.72 |
|  |  | C18:2 | 11.23±1.22 | 10.18±0.27 | 9.9±0.42 | 11.78±0.54 | 10.33±0.26 |
|  |  | C18:3 | 0.86±0.09 | 0.82±0.09 | 0.9±0.12 | 1.1±0.1 | 0.96±0.21 |
|  |  | C20:4 | 0.43±0.1 | 0.74±0.23 | 0.53±0.09 | 0.39±0.03 | 0.33±0.08 |
|  |  | C22:6 | 0.36±0.08 | 0.68±0.23 | 0.43±0.08 | 0.31±0.04 | 0.24±0.06 |
|  |  |  |  |  |  |  |  |
|  | 32% | C14:0 | 1.56±0.31 | 1.49±0.16 | 1.21±0.2 | 0.88±0.1 | 0.86±0.09 |
|  |  | C15:1 | 1.79±0.4 | 1.43±0.12 | 1.6±0.28 | 1.2±0.15 | 1.03±0.1 |
|  |  | C16:0 | 9.98±0.5 | 15±0.72 | 11.53±0.66 | 11.19±0.62 | 10.56±0.43 |
|  |  | C16:1 | 1.16±0.06 | 1.11±0.08 | 1.29±0.09 | 1.17±0.11 | 1.16±0.05 |
|  |  | C18:0 | 4.76±0.25 | 4.62±0.21 | 4.99±0.22 | 4.94±0.23 | 4.54±0.22 |
|  |  | C18:1 | 40.87±1.39 | 39.17±1.57 | 42.48±1.11 | 42.78±0.46 | 43.38±0.93 |
|  |  | C18:2 | 18.64±1.02 | 11.97±0.54 | 20.35±0.94 | 21.55±0.42 | 21.08±0.44 |
|  |  | C18:3 | 1.03±0.15 | 1.08±0.08 | 1.34±0.18 | 1.54±0.11 | 1.37±0.08 |
|  |  | C20:4 | 0.71±0.16 | 0.66±0.08 | 0.75±0.15 | 0.64±0.08 | 0.57±0.06 |
|  |  | C22:6 | 0.58±0.15 | 0.59±0.08 | 0.53±0.17 | 0.42±0.06 | 0.43±0.08 |
|  |  |  |  |  |  |  |  |
| Polar | 13% | C14:0 | 0.08±0.02 | 0.15±0.03 | 0.14±0.03 | 0.07±0.02 | 0.09±0.02 |
|  |  | C15:1 | 0.07±0.01 | 0.1±0.02 | 0.11±0.03 | 0.06±0.01 | 0.07±0.01 |
|  |  | C16:0 | 21.25±0.83 | 19.78±0.47 | 20.12±0.7 | 21.26±0.81 | 21.86±1.16 |
|  |  | C16:1 | 0.19±0.02 | 0.24±0.02 | 0.2±0.01 | 0.14±0.03 | 0.16±0.03 |
|  |  | C18:0 | 22.13±0.3 | 22.19±0.48 | 22.19±0.53 | 22.29±0.49 | 22.25±0.17 |
|  |  | C18:1 | 10.72±0.36 | 10.16±0.57 | 10.79±0.27 | 10.49±0.3 | 9.78±0.2 |
|  |  | C18:2 | 12.27±0.55 | 10.72±0.45 | 13.24±0.39 | 12.45±0.34 | 13.44±0.3 |
|  |  | C18:3 | 0.43±0.05 | 0.45±0.07 | 0.59±0.04 | 0.51±0.07 | 0.62±0.03 |
|  |  | C20:4 | 9.56±0.33 | 9.57±0.55 | 10.17±0.33 | 9.02±0.41 | 10.05±0.22 |
|  |  | C22:6 | 11.09±0.71 | 11.2±0.84 | 9.69±0.34 | 10.71±0.87 | 10.88±0.62 |
|  |  |  |  |  |  |  |  |
|  | 32% | C14:0 | 0.17±0.04 | 0.09±0.01 | 0.13±0.03 | 0.74±0.69 | 0.14±0.05 |
|  |  | C15:1 | 0.12±0.02 | 0.07±0 | 0.11±0.02 | 0.23±0.15 | 0.13±0.05 |
|  |  | C16:0 | 16.41±0.78 | 17.57±0.56 | 18.77±0.53 | 15.96±0.89 | 18.18±0.95 |
|  |  | C16:1 | 0.17±0.01 | 0.16±0.01 | 0.17±0 | 0.26±0.12 | 0.18±0.03 |
|  |  | C18:0 | 24.82±0.64 | 23.54±0.39 | 23.95±0.39 | 22.62±1.52 | 22.89±0.79 |
|  |  | C18:1 | 8.55±0.31 | 9.03±0.31 | 8.13±0.16 | 7.65±0.61 | 9.22±0.47 |
|  |  | C18:2 | 16.28±0.69 | 14.5±0.67 | 15.94±0.64 | 15.61±1.09 | 16.95±0.56 |
|  |  | C18:3 | 0.53±0.05 | 0.6±0.04 | 0.59±0.04 | 0.52±0.04 | 0.6±0.04 |
|  |  | C20:4 | 9.38±0.48 | 9.81±0.28 | 10.05±0.39 | 8.09±0.77 | 8.88±0.24 |
|  |  | C22:6 | 10.73±0.77 | 11.85±0.52 | 9.65±0.34 | 8.81±0.69 | 10.81±0.9 |

Figure C-1. The effects of dietary linoleic acid (18:2n6) and wind-tunnel flight training on the composition of palmitic acid (16:0, A) and 18:2n6 (B) in the lipid droplets of flight muscle in European Starlings. Palmitic acid also showed significant diet-specific changes in concentration over time (C). Composition is presented as the percent of total fatty acids by mass.

Figure C-2. The effects of dietary linoleic acid (18:2n6) and wind-tunnel flight training on the composition of palmitic acid (16:0, A), docosahexaenoic acid (22:6n3, B), stearic acid (18:0, C), and 18:2n6 (D) in the membranes of flight muscle in European Starlings. Stearic acid (E) and linoleic acid (F) also showed significant diet-independent changes in concentration over time. Composition is presented as the percent of total fatty acids by mass.
