# Supplementary material for: The effects of dietary linoleic acid and hydrophilic antioxidants on basal, peak, and sustained metabolism in flight‐trained European starlings
Source: Ecol Evol. 2020 Jan 18;10(3):1552–66. doi: 10.1002/ece3.6010 (PMC7029098; doi:10.1002/ece3.6010)
Supplement: Supplementary file 6 [file ECE3-10-1552-s006.docx]

*Appendix A: Study design*

Figure A-1. Schematic illustration of the experimental design of this study depicting the arrangement of cohorts over time, when birds were housed in different spaces and the timing different measurements and samples. Bars denote ongoing housing or procedures while Xs denote point procedures. Grey symbols indicate that all birds in the cohort were involved while white symbols involved only control birds and black symbols involved only trained birds.
